# Predictive Factors for Positive Surgical Margins in Patients With Prostate Cancer After Radical Prostatectomy: A Systematic Review and Meta-Analysis

**DOI:** 10.3389/fonc.2020.539592

**Published:** 2021-02-08

**Authors:** Lijin Zhang, Hu Zhao, Bin Wu, Zhenlei Zha, Jun Yuan, Yejun Feng

**Affiliations:** Department of Urology, Affiliated Jiang-yin Hospital of the Southeast University Medical College, Jiang-yin, China

**Keywords:** prostate cancer, radical prostatectomy, positive surgical margins, risk factors, meta-analysis

## Abstract

**Background and Objectives:**

Previous studies have demonstrated that positive surgical margins (PSMs) were independent predictive factors for biochemical and oncologic outcomes in patients with prostate cancer (PCa). This study aimed to conduct a meta-analysis to identify the predictive factors for PSMs after radical prostatectomy (RP).

**Methods:**

We selected eligible studies *via* the electronic databases, such as PubMed, Web of Science, and EMBASE, from inception to December 2020. The risk factors for PSMs following RP were identified. The pooled estimates of standardized mean differences (SMDs)/odds ratios (ORs) and 95% confidence intervals (CIs) were calculated. A fixed effect or random effect was used to pool the estimates. Subgroup analyses were performed to explore the reasons for heterogeneity.

**Results:**

Twenty-seven studies including 50,014 patients with PCa were eligible for further analysis. The results showed that PSMs were significantly associated with preoperative prostate-specific antigen (PSA) (pooled SMD = 0.37; 95% CI: 0.31–0.43; P < 0.001), biopsy Gleason Score (<6/≥7) (pooled OR = 1.53; 95% CI:1.31–1.79; P < 0.001), pathological Gleason Score (<6/≥7) (pooled OR = 2.49; 95% CI: 2.19–2.83; P < 0.001), pathological stage (<T2/≥T3) (pooled OR = 3.90; 95% CI: 3.18–4.79; P < 0.001), positive lymph node (PLN) (pooled OR = 3.12; 95% CI: 2.28–4.27; P < 0.001), extraprostatic extension (EPE) (pooled OR = 4.44; 95% CI: 3.25–6.09; P < 0.001), and seminal vesicle invasion (SVI) (pooled OR = 4.19; 95% CI: 2,87–6.13; P < 0.001). However, we found that age (pooled SMD = 0.01; 95% CI: −0.07–0.10; P = 0.735), body mass index (BMI) (pooled SMD = 0.12; 95% CI: −0.05–0.30; P = 0.162), prostate volume (pooled SMD = −0.28; 95% CI: −0.62–0.05; P = 0.097), and nerve sparing (pooled OR = 0.90; 95% CI: 0.71–1.14; P = 0.388) had no effect on PSMs after RP. Besides, the findings in this study were found to be reliable by our sensitivity and subgroup analyses.

**Conclusions:**

Preoperative PSA, biopsy Gleason Score, pathological Gleason Score, pathological stage, positive lymph node, extraprostatic extension, and seminal vesicle invasion are independent predictors of PSMs after RP. These results may helpful for risk stratification and individualized therapy in PCa patients.

## Introduction

Prostate cancer (PCa) is the most common type of newly diagnosed malignancy and a leading cause of cancer-related death in males worldwide (1). With the wide use of the prostate−specific antigen (PSA) screening test, the majority of PCa patients are diagnosed in the early stages (2). As a result, radical prostatectomy (RP) with bilateral pelvic lymph node dissection has been the gold standard for the treatment of patients with localized PCa (3). The goal of RP for PCa is complete prostate extirpation; despite favorable cancer control associated with RP, approximately 25% of all patients experience biochemical recurrence (BCR) (4). A number of factors have been found to be associated with BCR after RP, and one adverse risk factor is the presence of positive surgical margins (PSMs).

PSMs are defined as an extension of cancer cells to the inked cut surface of the RP specimen (5). Our previous findings have indicated that PSMs are significantly associated with BCR and poor survival outcome after RP (6, 7). However, none of the systematic research studies have reported about the factors that may affect the margin status of PCa after RP. Conventional parameters for risk estimation of PSMs are mainly based on factors, including preoperative PSA (p-PSA), pathological T stage, pathological Gleason Score (GS), and multiple positive biopsy cores (8–11). However, the prognostic value of these predictive factors is limited. Besides, PSMs may be affected by remnant normal tissue and inadequate surgical skill (12). Therefore, no consensus has been reported regarding the above results. Based on these considerations, a comprehensive meta-analysis and systematic review was necessary to evaluate the predictive factors for PSMs in PCa patients following RP.

## Materials and Methods

### Literature and Search Strategy

We carried out this meta-analysis in accordance with the guidelines of the Preferred Reporting Items for Systematic Review and Meta-Analyses statement (PRISMA) (13). A comprehensive literature search was conducted using the PubMed, Web of Science, Wanfang, and China National Knowledge Infrastructure (CNKI) databases. Search strategies were based on the combination of Medical Subject Headings (MeSH) and keywords as follows: “prostate cancer,” “radical prostatectomy,” “positive surgical margin,” “clinicopathological” and “risk factors.” The last search was conducted on December 2020. Meanwhile, to identify other eligible publications, reference lists were also screened manually. The language was restricted to English and Chinese. Because we did not perform clinical research in this study, no ethical approval was needed and all analyses were based on previously published literatures.

### Selection Criteria and Data Extraction

Papers were included in this meta-analysis if they met the following criteria: (1) all patients with a diagnosis of PCa and PSMs were histopathologically confirmed; (2) treatment was limited to RP; (3) clinicopathological features were analyzed according to the surgical margins status, and all studies had a comparable study design; (4) standardized mean differences (SMDs)/odds ratios (ORs) and 95% confidence intervals (CIs) were reported in the paper or could be computed from the given data; (5) if more than one article was identified in the same cohort, the most comprehensive and largest dataset was adopted. Accordingly, studies with the following criteria were excluded: (1) case reports, review articles, editorials, and non-original articles; (2) papers not published in English and Chinese; (3) studies that did not analyze the PSMs and clinical features; (4) studies lacking sufficient data to acquire SMDs/ORs and 95% CIs. Literature search was independently performed by two investigators. Disagreement was resolved by discussion.

### Data Extraction and Quality Assessment

Two researchers (BW and ZZ) assessed the titles and abstracts of the searched studies, respectively. Any disagreements were reconciled by a third researcher (JY). The following information was extracted from the included studies: publication information (first author’s last name, publication year, country of origin, and study design), patients’ characteristics (mean age, p-PSA, and follow-up time), and PCa outcomes (tumor stage, GS, and oncologic outcomes). According to the Newcastle–Ottawa quality assessment scale (NOS) (14), two researchers (HZ and YF) independently assessed the quality of each study. According to its criteria, the NOS estimates studies based on the following three parts: selection, comparability, and outcome assessment. For quality assessment, scores ranged from 0 to 9, and studies with scores of 6 or more were rated as being of high quality.

### Statistical Analysis

For this meta-analysis, pooled SMDs/ORs with 95% CIs were used to describe the relationship between risk factors and PSMs. An OR >1 or SMD >0 suggested a close relationship of PSMs in patients with PCa. Heterogeneity among studies was evaluated by using Cochran’s Q test and Higgins ***I***-squared statistic. If the ***I^2^*** value was >50% or the *P_heterogeneity_* was <0.1, it suggested a statistically significant heterogeneity in the included studies, and a random-effects (RE) model was adopted; otherwise, a fixed-effects (FE) model was used. To consider the potential reason for heterogeneity, subgroup analysis was conducted. To test the stability of the result, we performed a sensitivity analysis by excluding one study in turn. Visual inspection of asymmetry in funnel plots was carried out to assess the potential publication bias. Furthermore, we performed Egger’s tests to provide quantitative evidence of publication bias. These statistical analyses or data syntheses were calculated using STATA version 12.0 (Stata Corporation, College Station, TX, USA). All statistical tests were two sided, and P < 0.05 was considered statistically significant.

## Results

### Literature Search

**Figure 1:**
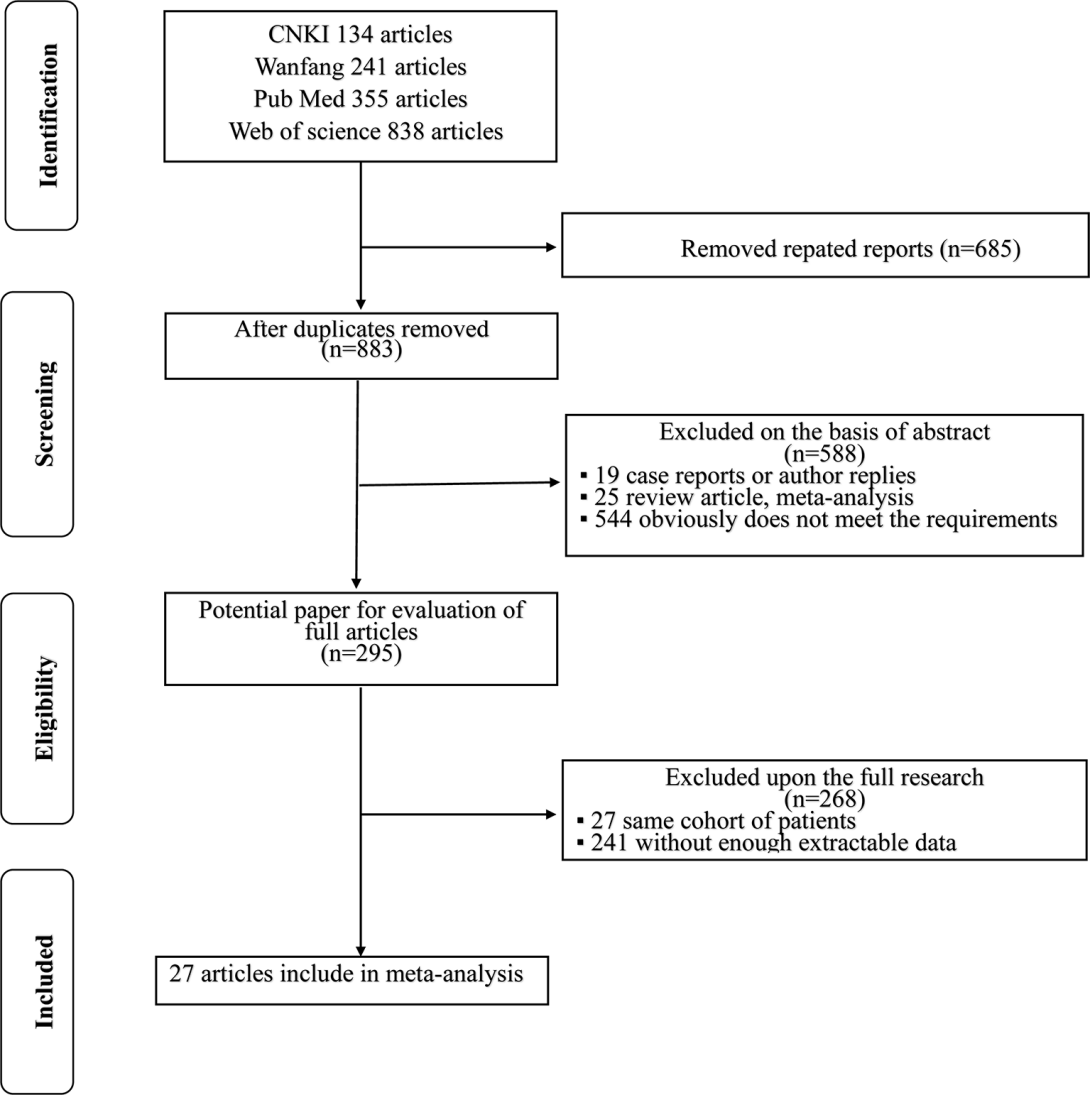
Flowchart of the literature review process for the selection of eligible literatures.

A flowchart of the literature selection process is shown in **Figure 1**. The initial search of electronic databases identified 1,568 records according to the search criteria; after the duplicates were removed, 883 papers remained behind. A total of 588 papers were then excluded by screening the titles and abstracts. Then, 295 full-text articles were further examined and 268 articles were excluded because 27 articles included the same cohort of patients and 241 articles lacked enough data for further research. Finally, 27 articles (8, 15–40) published between 2009 and 2020 were included in this meta-analysis.

### Features of the Included Studies

Summary of the major characteristics of these studies is shown in **Table 1** and **Table 2**. All studies had a retrospective study design. The sample size ranged from 144 to 12,515, and a total of 50,014 patients were included. A total of 12,093 PCa patients with PSMs were included in our study, which accounted for 24.2% of all patients. Geographically, eight studies were conducted in Asia, eight in North America, eight in Europe, two in Australia, and one in multi-center locations. All patients had received RP as primary treatment for PCa. According to the NOS quality assessment, all studies included in this study were categorized as being of high quality (**Supplementary Table S1**).

**Table 1:** The basic characteristics of all studies included in this meta-analysis.

Author	Year	Country	Recruitment period	No. of patients		Age (years)		Pre-PSA		Follow-up (months)	
				PSMs	NSMs	PSMs	NSMs	PSMs	NSMs	PSMs	NSMs
Celik et al. (15)	2020	Turkey	2005–2020	893	1,750	Mean ± SD 63.2 ± 6.5	Mean ± SD 62.4 ± 6.7	Mean ± SD 13 ± 18.9	Mean ± SD 8.8 ± 9.5	NA	NA
Porcaro et al. (16)	2020	Italy	2013–2017	192	540	Median (IQR) 65 (60–69)	Median (IQR) 65 (60–69)	Median (IQR) 6.9 (5.1–8.7)	Median (IQR) 6.1 (4.8–8.3)	Median (IQR) 26 (14–40)	Median (IQR) 26 (14–40)
Tian et al. (17)	2019	China	2010–2016	142	267	Median (IQR) 70 (62.8–75.0)	Median (IQR) 71 (66.0–75.0)	Median (IQR) 13.7 (9.3–25.0)	Median (IQR) 10.2 (6.7–17.7)	NA	NA
Martini et al. (18)	2019	Italy	2011–2017	285	1,472	Median (IQR) 64.8 (58.9–70.0)	Median (IQR) 64.6 (59.0–69.7)	Median (IQR) 7.2 (5.5–10.6)	Median (IQR) 6.3 (4.6–8.3)	Median 30	Median 30
Hou et al. (19)	2019	China	2007–2017	94	226	Median (IQR) 67.9 (45–80)	Median (IQR) 67.9 (45–80)	Median (IQR) 14.4 (1–123)	Median (IQR) 14.4 (1–123)	NA	NA
Herforth et al. (20)	2018	USA	1988–2015	1,902	2,063	Median (IQR) 62 (58–66)	Median (IQR) 63 (58–67)	Median (IQR) 7.5 (5.2–12)	Median (IQR) 5.9 (4.4–8.5)	Median (IQR) 93 (53–152)	Median (IQR) 105 (63–147)
Tatsugami et al. (21)	2017	Japan	2009–2013	594	1,794	Mean ± SD 64.9 ± 6.2	Mean ± SD 65.3 ± 6.2	Median (range) 6.6 (1.8–57.1)	Median (range) 7.7 (3.0–69.8)	Median (range) 9 (1–83)	Median (range) 9 (1–83)
Seo et al. (8)	2017	Korea	2008–2014	50	94	Mean ± SD 64.6 ± 6.5	Mean ± SD 67.3 ± 6.7	Mean ± SD 16.3 ± 11.4	Mean ± SD 10.5 ± 6.7	Mean ± SD 55.4 ± 3.9	Mean ± SD 64.1 ± 2.0
Meyer et al. (22)	2017	USA	1992–2005	118	785	Median (IQR) 63 (60–67)	Median (IQR) 63 (58–66)	Median (IQR) 6 (4.3–9.0)	Median (IQR) 6.4 (4.6–8.9)	Median (IQR) 132 (86–145)	Median (IQR) 133 (99–157)
Abdollah et al. (23)	2016	MC	2002–2013	1,045	11,470	Median (IQR) 62 (56–67)	Median (IQR) 61 (55–56)	Median (IQR) 6.2 (4.7–9.6)	Median (IQR) 5.2 (4.1–7.2)	Median 39	Median 39
Whalen et al. (24)	2015	USA	2005–2011	126	453	Mean ± SD 61.0 ± 7.7	Mean ± SD 61.3 ± 7.0	Mean ± SD 9.2 ± 8.6	Mean ± SD 6.1 ± 5.4	Median (range) 20.5 (1–80)	Median (range) 20.5 (1–80)
Retèl et al. (25)	2014	Switzerland	1990–2008	479	775	Mean ± SD 63.4 ± 6.0	Mean ± SD 62.9 ± 6.5	NA	NA	Median (range) 73.2 (2–120)	Median (range) 73.2 (2–120)
Rouanne et al. (26)	2014	France	1988–2001	108	295	Median (range) 66 (47–77)	Median (range) 66 (46–81)	Median (range) 10 (2–158)	Median (range) 10 (0.5–134)	Median (range) 139 (126–231)	Median (range) 147 (134–251)
Sammon et al. (27)	2013	USA	1993–2010	162	632	Mean ± SD 63.1 ± 8.9	Mean ± SD 63.5 ± 7.8	Mean ± SD 6.9 ± 4.6	Mean ± SD 5.3 ± 3.3	Median (IQR) 54 (27–84)	Median (IQR) 54 (27–84)
Lee et al. (28)	2013	Korea	2005–2011	167	200	Mean ± SD 67.9 ± 5.7	Mean ± SD 67.8 ± 5.3	Mean ± SD 11.2 ± 10.4	Mean ± SD 8.4 ± 6.4	NA	NA
Hashimoto et al. (29)	2013	Japan	2006–2011	54	190	Mean ± SD 64.8 ± 5.7	Mean ± SD 64.0 ± 6.8	Mean ± SD 12.5 ± 12.6	Mean ± SD 9.3 ± 7.3	NA	NA
Abdollah et al. (30)	2013	Italy	1998–2010	305	1,198	Median (range) 64.6 (40.5–81.1)	Median (range) 64.8 (42.3–82.2)	Median (range) 6.6 (1–74.1)	Median (range) 6.2 (0.2–47.8)	Mean 122.5	Mean 122.5
Savdie et al. (31)	2012	Australia	1997–2003	285	655	Median (range) 61.7 (46.4–81)	Median (range) 61.2 (42.2–77.4)	Median (range) 8.7 (2–63)	Median (range) 7.5 (0.4–84)	Median (range) 82 (5–146)	Median (range) 82 (5–146)
Lu et al. (32)	2012	China	1993–1999	250	544	Median (IQR) 62 (57–66)	Median (IQR) 62 (52–66)	Median (IQR) 6.2 (4.5–9.3)	Median (IQR) 5.9 (4.5–8.0)	Median (IQR) 115.2 (72–132)	Median (IQR) 120 (78–135.6)
Karavitakis et al. (33)	2012	UK	2007–2009	31	64	Mean 62.9	Mean 61.3	Mean 13.9	Mean 10.9	NA	NA
Corcoran et al. (34)	2012	Australia	1995–2010	370	1,144	Median (range) 61.5 (40.2–79.8)	Median (range) 61.5 (40.2–79.8)	Mean ± SD 7.8 ± 6.6	Mean ± SD 7.8 ± 6.6	Median (range) 22.2 (0.8–181)	Median (range) 22.2 (0.8–181)
Li et al. (35)	2011	China	2000–2009	57	92	Mean ± SD 70.2 ± 6.3	Mean ± SD 69.0 ± 6.0	Mean ± SD 13.4 ± 17.6	Mean ± SD 8.0 ± 5.8	Mean ± SD 46.8 ± 27.8	Mean ± SD 46.8 ± 27.8
Coelho et al. (36)	2010	USA	2008–2009	101	775	Median (IQR) 62 (56–66)	Median (IQR) 61 (56–66)	Median (IQR) 5 (3.9–6.9)	Median (IQR) 4.9 (3.8–6.6)	NA	NA
Boorjian et al. (37)	2010	USA	1990–2006	3,651	8,078	Median (IQR) 64 (59–69)	Median (IQR) 63 (57–68)	Median (IQR) 8.1 (5.4–14.1)	Median (IQR) 5.9 (4.1–8.7)	Median (IQR) 98.4 (52.8–145.2)	Median (IQR) 98.4 (52.8–145.2)
Alkhateeb et al. (38)	2010	Canada	1992–2008	264	1,004	Mean ± SD 62 ± 6.6	Mean ± SD 62 ± 6.6	Mean (range) 7.7 (0.1–65.9)	Mean (range) 7.7 (0.1–65.9)	Mean (range) 78.1 (3–192)	Mean (range) 78.1 (3–192)
Shikanov et al. (39)	2009	USA	2003–2008	243	1,155	Median (IQR) 59 (54–65)	Median (IQR) 60 (55–65)	Median (IQR) 5.6 (4.4–8.1)	Median (IQR) 5.1 (4.1–7.1)	Median (IQR) 12.3 (6.3–18.9)	Median (IQR) 12.3 (6.3–20.1)
Ficarra et al. (40)	2009	Italy	2005–2008	95	227	Mean 61.4	Mean 61.4	NA	NA	Median 14	Median 14

SD, standard deviation; NA, data not applicable; MC, Multi-centers; PSMs, positive surgical margins; NSMs, negative surgical margins.

**Table 2:** The main pathological characteristics of all studies included in this meta-analysis.

Author	Stagingsystem	Gradingsystem	Biopsy GS <6/≥7		Pathological GS <6/≥7		Pathological stage 1–2/3–4	
			PSMs	NSMs	PSMs	NSMs	PSMs	NSMs
Celik et al. (15)	TNM	2014 ISUP	NA	NA	NA	NA	427/466	1,377/413
Porcaro et al. (16)	2010 TNM	2014 ISUP	81/111	262/278	19/173	107/433	161/31	453/87
Tian et al. (17)	2012TNM	Gleason score	NA	NA	NA	NA	75/67	212/64
Martini et al. (18)	TNM	Gleason score	NA	NA	203/82	1,246/208	108/177	969/503
Hou et al. (19)	TNM	Gleason score	27/67	101/125	16/78	84/142	46/48	174/52
Herforth et al. (20)	TNM	Gleason score	NA	NA	NA	NA	1,249/653	1,567/496
Tatsugami et al. (21)	TNM	Gleason score	172/422	1,200/594	46/548	276/1,518	539/55	62/594
Seo et al. (8)	TNM	Gleason score	14/36	40/54	NA	NA	34/16	84/10
Meyer et al. (22)	2002TNM	Gleason score	98/20	625/120	69/49	510/275	NA	NA
Abdollah et al. (23)	TNM	Gleason score	436/891	1,726/2,237	138/1,198	1,167/2,796	373/954	2,883/1,080
Whalen et al. (24)	1997TNM	Gleason score	30/96	214/239	30/96	214/239	51/75	365/88
Retèl et al. (25)	TNM	Gleason score	NA	NA	224/255	502/273	239/240	629/146
Rouanne et al. (26)	TNM	Gleason score	81/27	233/62	49/59	181/114	35/73	224/71
Sammon et al. (27)	TNM	Gleason score	NA	NA	67/95	525/107	47/115	298/334
Lee et al. (28)	TNM	Gleason score	NA	NA	30/136	69/131	88/79	169/31
Hashimoto et al. (29)	NA	Gleason score	18/36	63/127	NA	NA	NA	NA
Abdollah et al. (30)	TNM	Gleason score	NA	NA	115/190	635/563	256/49	1,115/83
Savdie et al. (31)	TNM	Gleason score	NA	NA	75/210	241/414	105/180	438/217
Lu et al. (32)	TNM	Gleason score	NA	NA	80/170	293/251	161/89	468/76
Karavitakis et al. (33)	TNM	Gleason score	18/13	43/21	7/21	22/43	14/17	45/19
Corcoran et al. (34)	TNM	Gleason score	NA	NA	47/323	290/854	182/188	924/220
Li et al. (35)	1992TNM	Gleason score	NA	NA	NA	NA	NA	NA
Coelho et al. (36)	TNM	Gleason score	56/45	453/322	21/80	310/463	43/58	669/106
Boorjian et al. (37)	TNM	Gleason score	1,905/1,125	5,372/1,621	1,806/1,839	5,719/2,328	2,072/1,579	6,767/1,289
Alkhateeb et al. (38)	TNM	Gleason score	NA	NA	42/222	310/694	116/148	737/267
Shikanov et al. (39)	TNM	Gleason score	118/125	727/428	73/170	592/563	120/123	980/175
Ficarra et al. (40)	2002TNM	Gleason score	67/28	187/40	26/69	112/115	21/74	177/50

NA, data not applicable; PSMs, positive surgical margins; NSMs, negative surgical margins; GS, Gleason Score; ISUP, International Society of Urologic Pathology (ISUP) system.

### Meta-Analysis

**Figure 2:**
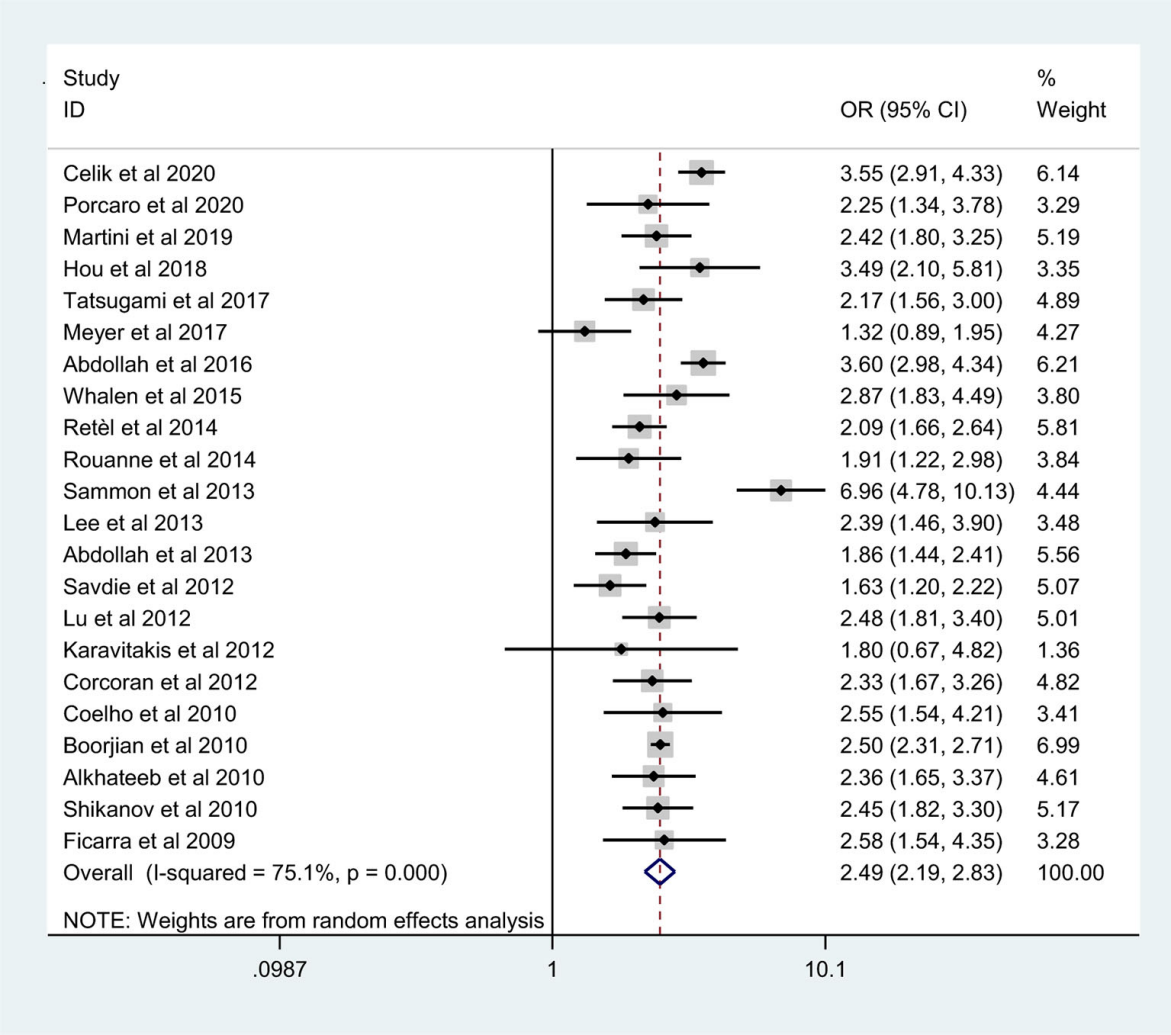
Forest plot for the association between pathological GS and PSMs risk.

**Figure 3:**
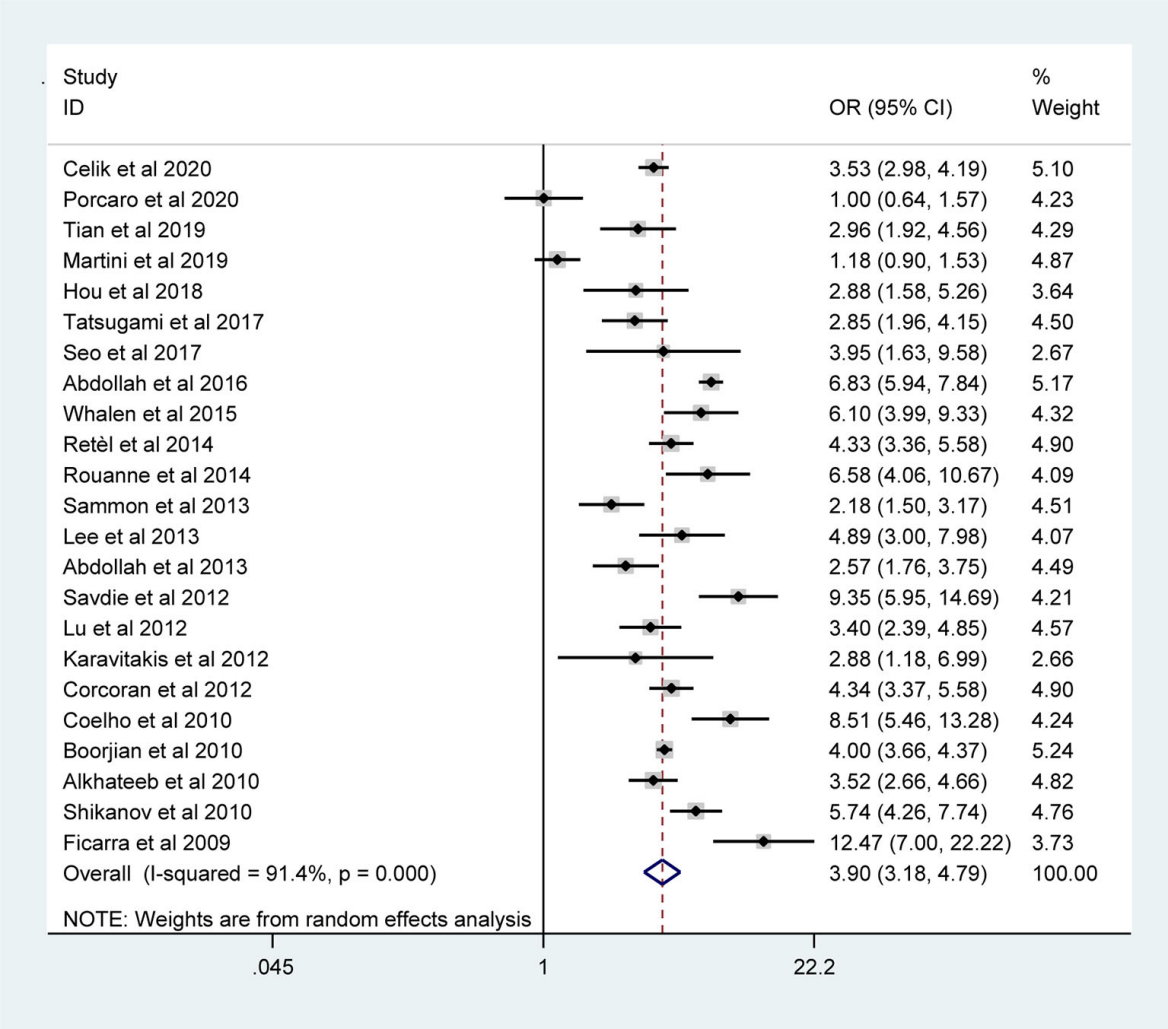
Forest plot reflecting the association between pathological stage and PSMs.

**Figure 4:**
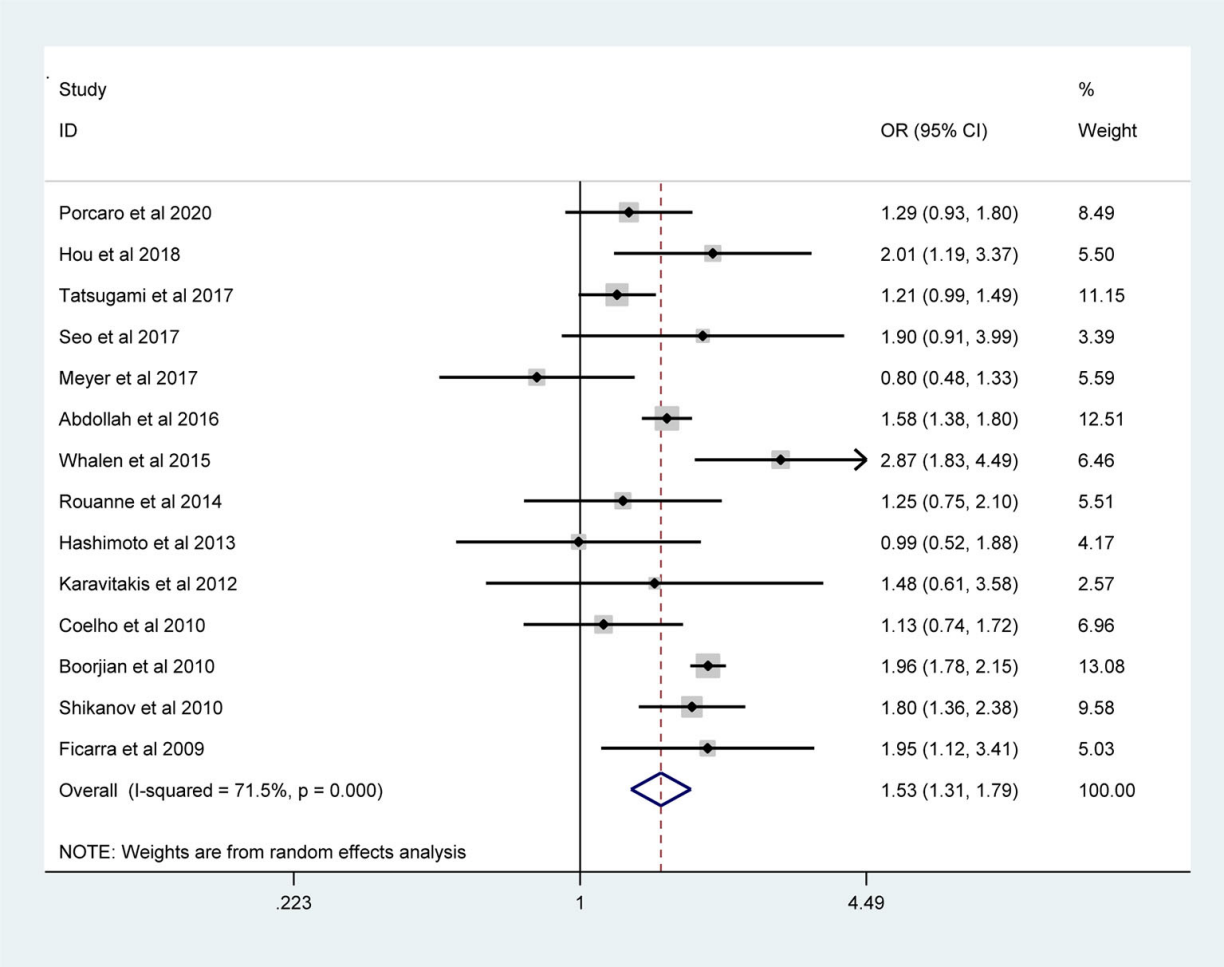
Forest plot assessing the correlation of biopsy GS and PSMs.

**Figure 5:**
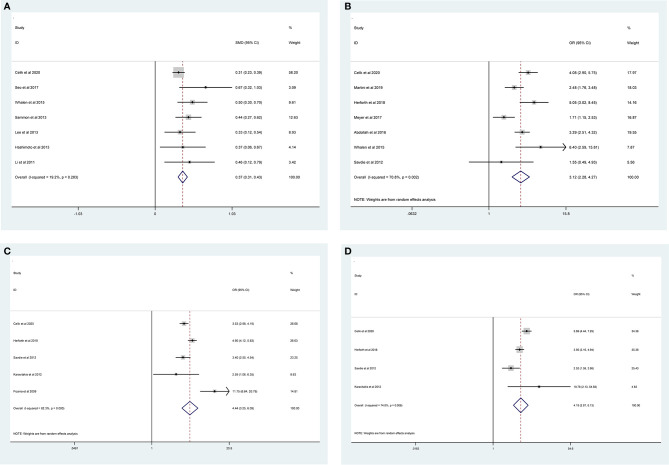
Forest plots of studies evaluating the prognostic factors for p-PSA **(A)**, PLN **(B)**, EPE **(C)**, and SVI **(D)** with PSMs risk.

The pooled results from the included studies indicated that PSMs were associated with pathological GS (<6/≥7) (RE model, pooled OR = 2.49; 95% CI: 2.19–2.83; P < 0.001, **Figure 2**), pathological stage (<T2/≥T3) (RE model, pooled OR = 3.90; 95% CI: 3.18–4.79; P < 0.001, **Figure 3**), biopsy GS (<6/≥7) (RE model, pooled OR = 1.53; 95% CI: 1.31–1.79; P < 0.001, **Figure 4**), p-PSA (FE model, pooled SMD = 0.37; 95% CI: 0.31–0.43; P < 0.001, **Figure 5A**), positive lymph node (PLN) (RE model, pooled OR = 3.12; 95% CI: 2.28–4.27; P < 0.001, **Figure 5B**), extraprostatic extension (EPE) (RE model, pooled OR = 4.44; 95% CI: 3.25–6.09; P < 0.001, **Figure 5C**), and seminal vesicle invasion (SVI) (RE model, pooled OR = 4.19; 95% CI: 2.87–6.13; P < 0.001, **Figure 5D**).

**Figure 6:**
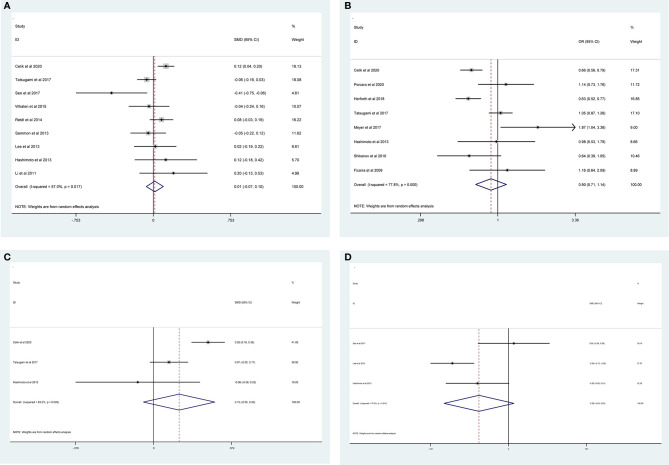
Forest plots of studies evaluating the association of PSMs and clinicopathological features in PCa patients. Age **(A)**, nerve sparing **(B)**, BMI **(C)**, and prostate volume **(D)**.

The results of meta-analysis of PSMs showed that no significant associations were found between PSMs and age (RE model, pooled SMD = 0.01; 95% CI: −0.07–0.10; P = 0.735, **Figure 6A**), nerve sparing (RE model, pooled OR = 0.90; 95% CI: 0.71–1.14; P = 0.388, **Figure 6B**), body mass index (BMI) (RE model, pooled SMD = 0.12; 95% CI: −0.05–0.30; P = 0.162, **Figure 6C**), and prostate volume (RE model, pooled SMD = −0.28; 95% CI: −0.62–0.05; P = 0.097, **Figure 6D**).

### Subgroup Analysis

Considering that there was no significant heterogeneity in p-PSA and the number of studies that evaluated BMI, SVI, and prostate volume was relatively small, we only conducted subgroup analysis for biopsy GS, pathological GS, pathological stage, PLN, EPE, age, and nerve sparing (**Table 3**). Subgroup analyses were conducted according to the geographical region (Asian *vs.* non-Asian), year of publication (≥2014 *vs.* <2014), number of patients (≥1,000 *vs.* <1,000), and median follow-up (≥70 months *vs.* <70 months). The results of subgroup analysis were roughly the same as overall results. Besides, the heterogeneity decreased significantly in some subgroup analyses, such as geographical region in Asian, year of publication <2014, and number of patients <1,000 cases.

**Table 3:** Summary and subgroup results for PSMs and clinicopathological features in PCa patients.

Analysis specification	No. of studies	Study heterogeneity		Effects model	Pooled OR/SMD (95% CI)	P-Value
		I^2^ (%)	P_heterogeneity_			
**BMI**						
Overall	3	83.2	0.003	Random	0.12 (–0.05,0.30)	0.162
**p-PSA**						
Overall	7	19.2	0.283	Fixed	0.37 (0.31,0.43)	<0.001
**SVI**						
Overall	4	74.8	0.008	Random	4.19 (2.87,6.13)	<0.001
**Prostate volume**						
Overall	3	76.3	0.015	Random	–0.28 (–0.62,0.05)	0.097
**Age**						
Overall	9	57	0.017	Random	0.01 (–0.07,0.10)	0.735
Geographical region						
Asian	5	49.6	0.094	Random	–0.03 (–0.17,0.12)	0.724
non-Asian	4	36.4	0.193	Fixed	0.06 (–0.02,0.14)	0.149
Year of publication						
≥2014	5	75.6	0.003	Random	–0.01 (–0.12,0.11)	0.916
<2014	4	0	0.543	Fixed	0.02 (–0.09,0.14)	0.675
No. of patients						
≥1,000	3	78.4	0.010	Random	0.05 (–0.07,0.16)	0.442
<1,000	6	34.0	0.182	Fixed	–0.02 (–0.14,0.10)	0.719
**Biopsy GS (<6/≥7)**						
Overall	14	71.5	<0.001	Random	1.53 (1.31,1.79)	<0.001
Geographical region						
Asian	4	28.2	0.243	Fixed	1.19 (0.90,1.58)	0.227
non-Asian	10	64.1	0.003	Random	1.65 (1.42,1.93)	<0.001
Year of publication						
≥2014	9	64.8	0.004	Random	1.44 (1.17,1.76)	<0.001
<2014	5	41.1	0.147	Fixed	1.75 (1.44,2.11)	<0.001
No. of patients						
≥1,000	5	50.2	0.090	Random	1.84 (1.40,2.42)	<0.001
<1,000	10	78.5	<0.001	Random	1.39 (1.13,1.70)	0.001
Median follow-up						
≥70 months	3	29.9	0.240	Fixed	1.58 (1.32,1.90)	<0.001
<70 months	6	68.1	0.008	Random	1.67 (1.13,2.46)	0.010
**P-GS (<6/≥7)**						
Overall	22	75.1	<0.001	Random	2.49 (2.19,2.83)	<0.001
Geographical region						
Asian	4	0	0.489	Fixed	2.47 (2.04,2.99)	<0.001
non-Asian	18	79.2	<0.001	Random	2.48 (2.14,2.89)	<0.001
Year of publication						
≥2014	9	74.3	<0.001	Random	2.37 (1.90,2.96)	<0.001
<2014	12	73.5	<0.001	Random	2.48 (2.08,2.95)	<0.001
No. of patients						
≥1,000	10	77.4	<0.001	Random	2.49 (2.02,3.07)	<0.001
<1,000	12	73.5	<0.001	Random	2.48 (2.08,2.95)	<0.001
Median follow-up						
≥70 months	8	66.2	0.004	Random	2.04 (1.74,2.39)	<0.001
<70 months	9	76.6	<0.001	Random	2.87 (2.27,3.62)	<0.001
**Stage (<T2/≥T3)**						
Overall	23	91.4	<0.001	Random	3.90 (3.18,4.79)	<0.001
Geographical region						
Asian	6	0	0.592	Fixed	3.32 (2.75,4.00)	<0.001
non-Asian	17	93.9	<0.001	Random	4.08 (3.19,5.22)	<0.001
Year of publication						
≥2014	11	94.8	<0.001	Random	3.28 (2.20,4.89)	<0.001
<2014	12	82.5	<0.001	Random	4.53 (3.64,5.64)	<0.001
No. of patients						
≥1,000	10	94.3	<0.001	Random	3.58 (2.74,4.69)	<0.001
<1,000	13	87.9	<0.001	Random	4.24 (2.88,6.25)	<0.001
Median follow-up						
≥70 months	7	75.8	<0.001	Random	4.24 (3.42,5.26)	<0.001
<70 months	10	95.8	<0.001	Random	3.58 (2.20,5.82)	<0.001
**Nerve sparing**						
Overall	8	77.8	<0.001	Random	0.90 (0.71,1.14)	0.388
Geographical region						
Asian	2	0	0.836	Fixed	1.04 (0.87,1.24)	0.666
non-Asian	6	74.8	0.001	Random	0.86 (0.65,1.14)	0.288
Year of publication						
≥2014	5	86.1	<0.001	Random	0.91 (0.67,1.24)	0.564
<2014	3	20.6	0.284	Fixed	0.87 (0.60,1.25)	0.452
No. of patients						
≥1,000	4	83.1	0.001	Random	0.74 (0.56,1.00)	0.06
<1,000	4	0	0.439	Fixed	1.23 (0.94,1.61)	0.130
Median follow-up						
≥70 months	2	91.7	0.001	Random	1.05 (0.36,3.05)	0.933
<70 months	4	22.0	0.279	Fixed	1.00 (0.81,1.23)	0.990
**EPE**						
Overall	5	82.3	0.001	Random	4.44 (3.25,6.09)	<0.001
Year of publication						
≥2014	2	85.6	0.008	Random	4.16 (3.02,5.74)	<0.001
<2014	3	87.2	<0.001	Random	4.80 (1.97,11.68)	0.001
No. of patients						
≥1,000	2	85.6	0.008	Random	4.16 (3.02,5.74)	<0.001
<1,000	3	87.2	<0.001	Random	4.80 (1.97,11.68)	0.001
**PLN**						
Overall	7	70.8	0.002	Random	3.12 (2.28,4.27)	<0.001
No. of patients						
≥1,000	4	56.4	0.076	Random	3.43 (2.66,4.54)	<0.001
<1,000	3	72.0	0.028	Random	2.52 (1.06,5.99)	0.037
Median follow-up						
≥70 months	3	82.8	0.003	Random	2.49 (1.07,5.79)	0.033
<70 months	3	53.7	0.115	Random	3.18 (2.24,4.52)	<0.001

### Sensitivity Analysis

To validate the reliability of our results, sensitivity analysis was performed. As shown in **Supplementary Figure S1**, the combined ORs for biopsy GS ranged from 1.47 (95% CI: 1.25 –1.72) to 1.58 (95% CI: 1.37–1.85) (**Supplementary Figure S1A**), the combined ORs for pathological GS ranged from 2.39 (95% CI: 2.14–2.67) to 2.56 (95% CI: 2.26–2.90) (**Supplementary Figure S1B**), the combined ORs for pathological stage ranged from 3.73 (95% CI: 3.04–4.58) to 4.15 (95% CI: 3.47–4.96) (**Supplementary Figure S1C**), the combined ORs for PLN ranged from 2.88 (95% CI: 2.08–4.00) to 3.51 (95% CI: 2.67–4.79) (**Supplementary Figure S1D**), the combined ORs for nerve sparing ranged from 0.83 (95% CI: 0.66–1.04) to 0.97 (95% CI: 0.74–1.27) (**Supplementary Figure S1E**), and the combined ORs for EPE ranged from 3.84 (95% CI: 3.05–4.85) to 4.68 (95% CI: 3.36–6.53) (**Supplementary Figure S1F**). The pooled SMD for p-PSA ranged from 0.36 (95% CI: 0.29–0.42) to 0.44 (95% CI: 0.35–0.54) (**Supplementary Figure S2A**), and the pooled SMD for age ranged from −0.01 (95% CI: −0.09–0.07) to 0.03 (95% CI: −0.05–0.12) (**Supplementary Figure S2B**). These data suggested that the results were statistically robust. Because the number of included studies for BMI, EPE, SVI, and prostate volume were small, the sensitivity analysis was not valuable.

### Publication Bias

**Figure 7:**
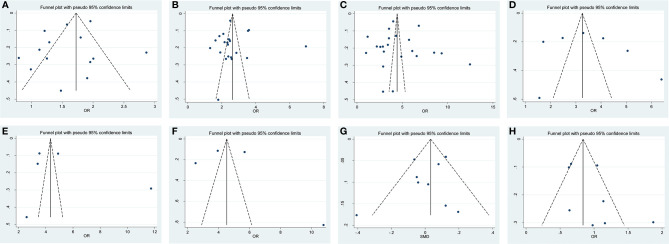
Funnel plot and Begg test for publication bias. **(A)** biopsy Gleason Score, **(B)** pathological GS, **(C)** pathological stage, **(D)** PLN, **(E)** EPE, **(F)** SVI, **(G)** age and **(H)** nerve sparing.

The shape of funnel plots did not reveal any evidence of asymmetry (**Figure 7**). The statistical results of Egger’s test still did not show any publication bias for biopsy GS (p- Egger = 0.277, **Figure 7A**), pathological GS (p- Egger = 0.945, **Figure 7B**), pathological stage (p- Egger = 0.830, **Figure 7C**), PLN (p- Egger = 0.605, **Figure 7D**), EPE (p- Egger = 0.513, **Figure 7E**), SVI (p- Egger = 0.797, **Figure 7F**), age (p- Egger = 0.431, **Figure 7G**), and nerve sparing (p- Egger = 0.197, **Figure 7H**). However, a minimal publication bias existed in p-PSA (p- Egger = 0.047). As the number of studies on prostate volume and BMI was limited, the publication bias was not assessed.

## Discussion

PSMs are unfavorable pathological features, which suggest incomplete tumor resection and confer poorer cancer control after RP (38). It was reported that PSMs were present in 11–38% of patients treated by RP and patients with PSMs have a higher risk of BCR compared to those with negative surgical margins (NSMs) (41). A multi-institutional review in 2009 conducted by Yossepowitch et al. (42) concluded that PSMs in RP specimens may be considered as an adverse outcome following RP. Consistent with these findings, our recent studies (6, 7) demonstrated the adverse effect of PSMs on both BCR and cancer-specific survival through a systematic review and meta-analysis. However, not all patients with PSMs have poor tumor outcomes, and some patients with localized PCa will show tumor progression even in the no-PSMs cases.

PSMs are factors that may be modified by the surgical technique. It seems that surgeon’s experience plays an important role in the decrease in the incidence of PSMs (43). Considerable efforts have been devoted to identifying factors, such as p-PSA (44), positive biopsy cores (10), and clinical stage (36), which can predict PSMs and clinical outcome following RP. The conclusion of several published studies indicated that several unfavorable pathological features may be associated with PSMs. However, inconsistent results have also been demonstrated in the published studies. Besides, for patients with adverse features of PSMs, prediction parameters that are currently available for PSMs may not reliable.

A retrospective study conducted by Boorjian et al. (37) found that increased p-PSA and BMI, higher pathological stage/GS, and greater tumor volume were significantly associated with the risk of PSMs. Likewise, Ficarra et al. (40) found an association between PSMs and biopsy GS, pathologic stage and GS, and EPE; however, no correlation was found between PSMs and p-PSA. Hashimoto et al. (29) found that only PSA density and prostate volume were independent predictors of PSMs after robot-assisted RP based on the data from 244 Japanese patients. Moreover, Yuksel et al. (45) considered the number of positive biopsies, pathologic stage and GS, SVI, and EPE as predictive factors for PSMs after robot-assisted RP. Meanwhile, no correlation was found with p-PSA, biopsy GS, and PLN. The inconsistent results from the above studies may due to small sample size, single-center design, and inhomogeneous population.

To the best of our knowledge, none of the studies have systematically addressed the preoperative predictive factors for PSMs after RP. In the present study, we identified 27 studies involving 50,014 patients, and the rate of PSMs was 24.2%, which is comparable to that in previous reports. The meta-analysis showed that p-PSA, biopsy GS (<6/≥7), pathological GS (<6/≥7), pathological stage (<T2/≥T3), PLN, EPE, and SVI had a statistically significant association with PSMs. Moreover, the pooled OR/SMD of the results suggested that age, BMI, prostate volume, and nerve sparing were not independent prognostic factors for PSMs in patients after RP. Subgroup analyses revealed a similar result despite different geographical regions, publication years, sample sizes, and median follow-ups. Further, sensitivity analysis and publication bias test were also performed, and the overall results showed that our data were stable and reliable.

This is the first comprehensive study to investigate the pathological features of PSMs and predictive factors for PSMs in patients treated with RP, and the results of this analysis are meaningful. The two strengths of this study are as follows: First, a large sample size of PCa patients from different geographic areas was included, and the findings of our study were more robust than those of an individual study. Second, a summary OR/SMD was conducted to compare the difference between PSMs and NSMs in PCa patients categorized by several confounders. Therefore, our findings could provide solid evidence for prognostic factors in PCa patients with PSMs.

Nevertheless, the present study has some limitations that should be acknowledged. First, all the studies were retrospectively performed, which made our research more susceptible to recall or selection bias. Second, a substantial heterogeneity was detected, while sensitivity analysis and subgroup analysis failed to identify the potential heterogeneity. Third, this study was limited to articles published in English and Chinese, which might have contributed to selection bias. As known, articles with positive results are more likely to be published. Therefore, this article also had a certain publication bias. Fourth, the number of included studies was limited in terms of publication bias and subgroup and sensitivity analyses, which could have led to unpersuasive conclusions. Therefore, more studies are required, which can provide more detailed individual high-quality data.

## Conclusion

The meta-analysis demonstrates that p-PSA, biopsy GS, pathological GS, pathological stage, PLN, EPE, and SVI were independent factors predicting PSMs after RP, and a combination of these factors might be useful for predicting PSMs in PCa patients undergoing RP. Considering the limitations of the present analysis, it is necessary to conduct more large-scale and well-designed studies to validate our results in the future.

## Data Availability Statement

All datasets generated for this study are included in the article/**Supplementary Material**.

## Author Contributions

LZ conceptualized the study. BW, ZZ, and JY performed the literatue search. HZ and YF analyzed the data. HZ wrote the original draft. LZ wrote, reviewed, and edited the manuscript. All authors contributed to the article and approved the submitted version.

## Conflict of Interest

The authors declare that the research was conducted in the absence of any commercial or financial relationships that could be construed as a potential conflict of interest.

## Supplementary Material

Supplementary Figure 1 Sensitivity analysis (pooled ORs) of the association between the predictive factors and PSMs risk. **(A)** biopsy GS; **(B)** pathological GS; **(C)** pathological stage; **(D)** PLN, and **(E)** nerve sparing.

Supplementary Figure 2 Sensitivity analysis (pooled SMDs) of the association between the predictive factors and PSMs risk. **(A)** p-PSA; **(B)** age.

## Abbreviations

PCa, renal cell cancer; PSMs, positive surgical margins; NSMs, negative surgical margins; RP, radical prostatectomy; PRISMA, Preferred Reporting Items for Systematic Reviews and Meta-Analyses; NOS, Newcastle Ottawa scale; ORs, odds ratios; SMD, standard mean differences; CIs, corresponding confidence intervals; p-PSA, preoperative PSA; GS, Gleason Score; PLN, positive lymph node; EPE, extraprostatic extension; SVI, seminal vesicle invasion; BMI, body mass index; RE, random-effects; FE, fixed-effects.
