# Supplementary figures and images for: Predictive Factors for Positive Surgical Margins in Patients With Prostate Cancer After Radical Prostatectomy: A Systematic Review and Meta-Analysis
Source: Front Oncol. 2021 Feb 8;10:539592. doi: 10.3389/fonc.2020.539592 (PMC7897672; doi:10.3389/fonc.2020.539592)

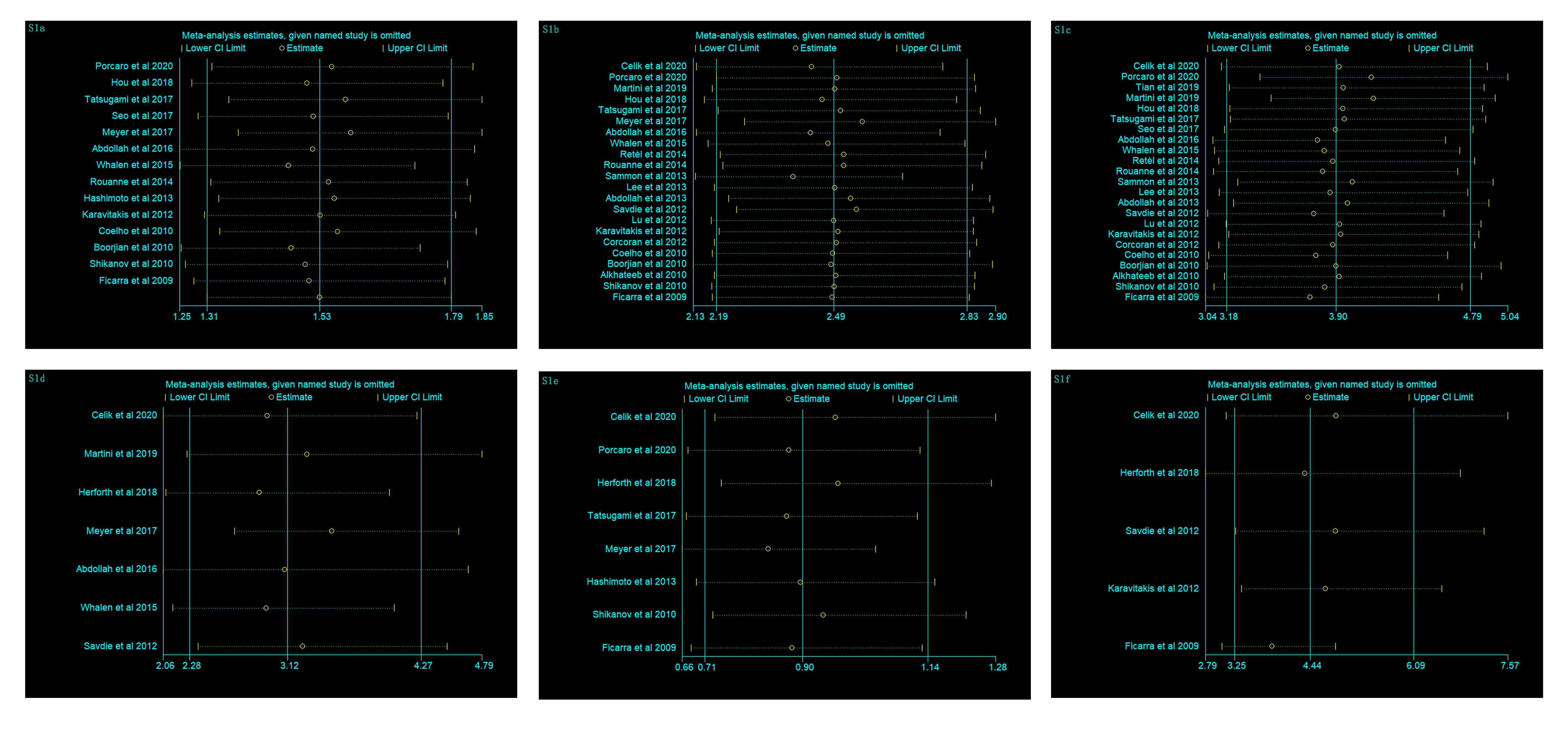

Supplement: Supplementary Figure 1 — Sensitivity analysis (pooled ORs) of the association between the predictive factors and PSMs risk. (A) biopsy GS; (B) pathological GS; (C) pathological stage; (D) PLN, and (E) nerve sparing. [file Image_1.tif]

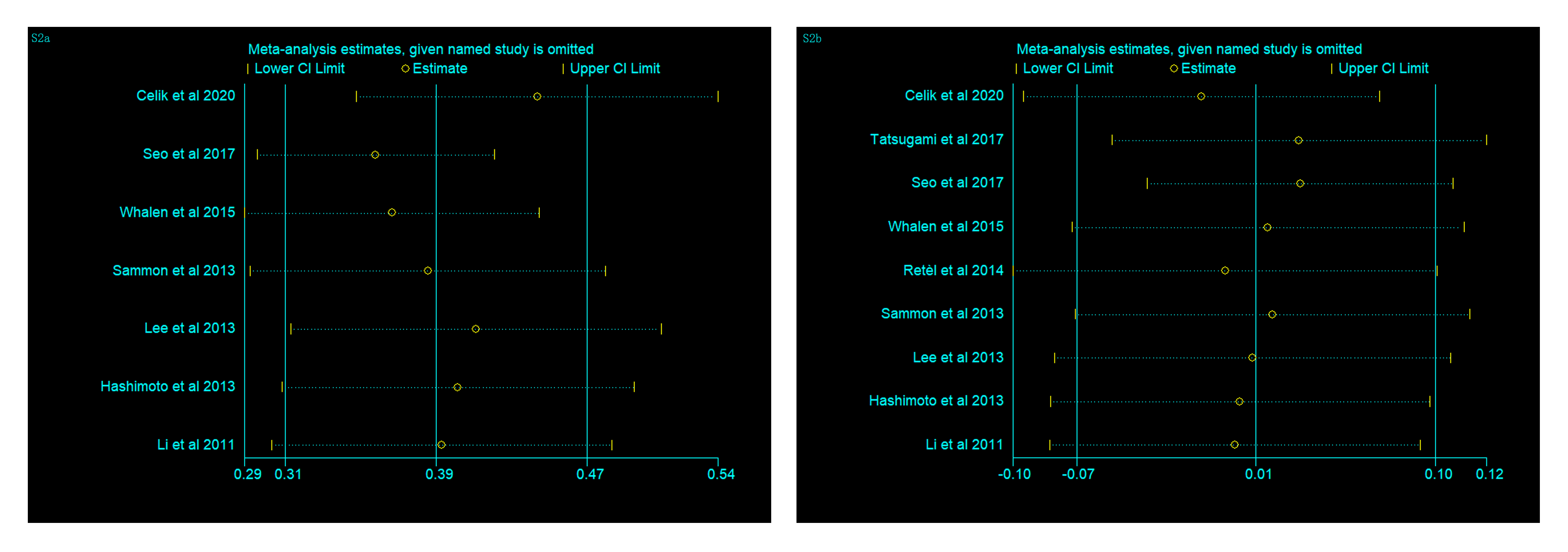

Supplement: Supplementary Figure 2 — Sensitivity analysis (pooled SMDs) of the association between the predictive factors and PSMs risk. (A) p-PSA; (B) age. [file Image_2.tif]
