# Supplementary material for: Predictive Factors for Positive Surgical Margins in Patients With Prostate Cancer After Radical Prostatectomy: A Systematic Review and Meta-Analysis
Source: Front Oncol. 2021 Feb 8;10:539592. doi: 10.3389/fonc.2020.539592 (PMC7897672; doi:10.3389/fonc.2020.539592)
Supplement: Supplementary file 3 [file Table_1.docx]

**Table S1. Quality assessment of cohort studies included in this meta- analysis**

| **Study** | **Representativeness of the exposed cohort** | **Selection of the unexposed cohort** | **Ascertainment of exposure** | **Outcome of interest not present at start of study** | **Control for important factor or additional factor** | **Outcome assessment** | **Follow-up long enough for outcomes to occur** | **Adequacy of follow-up of cohort** | **Total quality scores** |
| --- | --- | --- | --- | --- | --- | --- | --- | --- | --- |
| Celik et al[^15^](file:///C:\Users\张立进\Desktop\一审修回\Revised%20manuscript.docx#_ENREF_15) | ★ | ★ | ★ | ★ | ★ | ★ | ★ | — | 7 |
| Porcaro et al[^16^](file:///C:\Users\张立进\Desktop\一审修回\Revised%20manuscript.docx#_ENREF_16) | ★ | ★ | ★ | ★ | ★ | ★ | ★ | ★ | 8 |
| Tian et al[^17^](file:///C:\Users\张立进\Desktop\一审修回\Revised%20manuscript.docx#_ENREF_17) | ★ | ★ | ★ | ★ | ★ | ★ | — | — | 6 |
| Martini et al[^18^](file:///C:\Users\张立进\Desktop\一审修回\Revised%20manuscript.docx#_ENREF_18) | ★ | ★ | ★ | ★ | ★ | ★ | ★ | ★ | 8 |
| Hou et al[^19^](file:///C:\Users\张立进\Desktop\一审修回\Revised%20manuscript.docx#_ENREF_19) | ★ | ★ | ★ | ★ | ★ | ★ | — | — | 6 |
| Herforth et al[^20^](file:///C:\Users\张立进\Desktop\一审修回\Revised%20manuscript.docx#_ENREF_20) | ★ | ★ | ★ | ★ | ★ | ★ | ★ | ★ | 8 |
| Tatsugami et al[^21^](file:///C:\Users\张立进\Desktop\一审修回\Revised%20manuscript.docx#_ENREF_21) | ★ | ★ | ★ | ★ | ★ | ★ | ★ | — | 7 |
| Seo et al[^8^](file:///C:\Users\张立进\Desktop\一审修回\Revised%20manuscript.docx#_ENREF_8) | ★ | ★ | ★ | ★ | ★ | ★ | ★ | — | 7 |
| Meyer et al[^22^](file:///C:\Users\张立进\Desktop\一审修回\Revised%20manuscript.docx#_ENREF_22) | ★ | ★ | ★ | ★ | ★ | ★ | ★ | ★ | 8 |
| Abdollah et al[^23^](file:///C:\Users\张立进\Desktop\一审修回\Revised%20manuscript.docx#_ENREF_23) | ★ | ★ | ★ | ★ | ★★ | ★ | ★ | ★ | 9 |
| Whalen et al[^24^](file:///C:\Users\张立进\Desktop\一审修回\Revised%20manuscript.docx#_ENREF_24) | ★ | ★ | ★ | ★ | ★ | ★ | ★ | ★ | 8 |
| Retèl et al[^25^](file:///C:\Users\张立进\Desktop\一审修回\Revised%20manuscript.docx#_ENREF_25) | ★ | ★ | ★ | ★ | ★ | ★ | ★ | ★ | 8 |
| Rouanne et al[^26^](file:///C:\Users\张立进\Desktop\一审修回\Revised%20manuscript.docx#_ENREF_26) | ★ | ★ | ★ | ★ | ★ | ★ | ★ | ★ | 8 |
| Sammon et al[^27^](file:///C:\Users\张立进\Desktop\一审修回\Revised%20manuscript.docx#_ENREF_27) | ★ | ★ | ★ | ★ | ★ | ★ | ★ | ★ | 8 |
| Lee et al[^28^](file:///C:\Users\张立进\Desktop\一审修回\Revised%20manuscript.docx#_ENREF_28) | ★ | ★ | ★ | ★ | ★ | ★ | — | — | 6 |
| Hashimoto et al[^29^](file:///C:\Users\张立进\Desktop\一审修回\Revised%20manuscript.docx#_ENREF_29) | ★ | ★ | ★ | ★ | ★ | ★ | — | — | 6 |
| Abdollah et al[^30^](file:///C:\Users\张立进\Desktop\一审修回\Revised%20manuscript.docx#_ENREF_30) | ★ | ★ | ★ | ★ | ★ | ★ | ★ | ★ | 8 |
| Savdie et al[^31^](file:///C:\Users\张立进\Desktop\一审修回\Revised%20manuscript.docx#_ENREF_31) | ★ | ★ | ★ | ★ | ★★ | ★ | ★ | ★ | 9 |
| Lu et al[^32^](file:///C:\Users\张立进\Desktop\一审修回\Revised%20manuscript.docx#_ENREF_32) | ★ | ★ | ★ | ★ | ★ | ★ | ★ | ★ | 8 |
| Karavitakis et al[^33^](file:///C:\Users\张立进\Desktop\一审修回\Revised%20manuscript.docx#_ENREF_33) | ★ | ★ | ★ | ★ | ★ | ★ | — | — | 6 |
| Corcoran et al[^34^](file:///C:\Users\张立进\Desktop\一审修回\Revised%20manuscript.docx#_ENREF_34) | ★ | ★ | ★ | ★ | ★★ | ★ | ★ | ★ | 9 |
| Li et al[^35^](file:///C:\Users\张立进\Desktop\一审修回\Revised%20manuscript.docx#_ENREF_35) | ★ | ★ | ★ | ★ | — | ★ | ★ | ★ | 7 |
| Coelho et al[^36^](file:///C:\Users\张立进\Desktop\一审修回\Revised%20manuscript.docx#_ENREF_36) | ★ | ★ | ★ | ★ | ★ | ★ | — | — | 6 |
| Boorjian et al[^37^](file:///C:\Users\张立进\Desktop\一审修回\Revised%20manuscript.docx#_ENREF_37) | ★ | ★ | ★ | ★ | ★ | ★ | ★ | ★ | 8 |
| Alkhateeb et al[^38^](file:///C:\Users\张立进\Desktop\一审修回\Revised%20manuscript.docx#_ENREF_38) | ★ | ★ | ★ | ★ | ★ | ★ | ★ | ★ | 8 |
| Shikanov et al[^39^](file:///C:\Users\张立进\Desktop\一审修回\Revised%20manuscript.docx#_ENREF_39) | ★ | ★ | ★ | ★ | ★ | ★ | ★ | ★ | 8 |
| Ficarra et al^[40](file:///C:\\Users\\张立进\\Desktop\\一审修回\\Revised%20manuscript.docx#_ENREF_40)^ | ★ | ★ | ★ | ★ | ★ | ★ | — | ★ | 7 |
